# Evaluation of the FilmArray^®^ Pneumonia *Plus* Panel for Rapid Diagnosis of Hospital-Acquired Pneumonia in Intensive Care Unit Patients

**DOI:** 10.3389/fmicb.2020.02080

**Published:** 2020-08-25

**Authors:** Lise Crémet, Benjamin Gaborit, Marwan Bouras, Thomas Drumel, Florian Guillotin, Cécile Poulain, Elise Persyn, Karim Lakhal, Bertrand Rozec, Marie-Anne Vibet, Antoine Roquilly, Sophie Gibaud

**Affiliations:** ^1^Service de Bactériologie-Hygiène, Pôle de Biologie, CHU Nantes, Nantes, France; ^2^Laboratoire UPRES EA3826, IRS2 – Nantes Biotech, Université de Nantes, Nantes, France; ^3^Service de Maladies Infectieuses et Tropicales et CIC 1413, CHU Nantes, Nantes, France; ^4^Service d’Anesthésie Réanimation Chirurgicale, Pôle Anesthésie-Réanimation, CHU Nantes, Nantes, France; ^5^Service de Réanimation en Chirurgie Polyvalente, Pôle Anesthésie-Réanimation, Hôpital Nord Laennec, CHU Nantes, Nantes, France; ^6^Service de Réanimation en Chirurgie Cardio-Thoracique et Vasculaire, Pôle Anesthésie-Réanimation, Hôpital Nord Laennec, CHU Nantes, Nantes, France; ^7^Plateforme de Méthodologie et Biostatistique, CHU Nantes, Nantes, France

**Keywords:** multiplex syndromic testing, hospital-acquired pneumonia, rapid diagnosis, coinfection, antibiotic resistance

## Abstract

The FilmArray^®^ Pneumonia *plus* Panel (FAPP) is a new multiplex molecular test for hospital-acquired pneumonia (HAP), which can rapidly detect 18 bacteria, 9 viruses, and 7 resistance genes. We aimed to compare the diagnosis performance of FAPP with conventional testing in 100 intensive care unit (ICU) patients who required mechanical ventilation, with clinically suspected HAP. A total of 237 samples [76 bronchoalveolar lavages (BAL_DS_) and 82 endotracheal aspirates (ETA_DS_) obtained at HAP diagnosis, and 79 ETA obtained during follow-up (ETA_TT_)], were analyzed independently by routine microbiology testing and FAPP. 58 patients had paired BAL_DS_ and ETA_DS_. The positivity thresholds of semi-quantified bacteria were 10^3^–10^4^ CFUs/mL or 10^4^ copies/mL for BAL, and 10^5^ CFUs/mL or copies/mL for ETA. Respiratory commensals (*H. influenzae*, *S. aureus*, *E. coli*, *S. pneumoniae*) were the most common pathogens. Discordant results for bacterial identification were observed in 33/76 (43.4%) BAL_DS_ and 36/82 (43.9%) ETA_DS_, and in most cases, FAPP identified one supplemental bacteria (23/33 BAL_DS_ and 21/36 ETA_DS_). An absence of growth, or polybacterial cultures, explained almost equally the majority of the non-detections in culture. No linear relationship was observed between bin and CFUs/mL variables. Concordant results between paired BAL_DS_ and ETA_DS_ were obtained in 46/58 (79.3%) patients with FAPP. One of the 17 resistance genes detected with FAPP (*mecA/C* and MREJ) was not confirmed by conventional testing. Overall, FAPP enhanced the positivity rate of diagnostic testing, with increased recognition of coinfections. Implementing this strategy may allow clinicians to make more timely and informed decisions.

## Introduction

Hospital-acquired pneumonia (HAP) is the most frequent cause of nosocomial infection in intensive care unit (ICU) patients, with dramatic effects on patients’ outcomes. International experts have developed guidelines to prevent and improve the management of HAP (Kalil et al., 2016; Torres et al., 2017). Among strategies proposed, optimization of empiric antimicrobial therapy is of major importance. This entails administrating early appropriate antimicrobial therapy, while limiting overuse of broad-spectrum antibiotics. Hence, European guidelines suggest using narrow-spectrum empiric therapy (amoxicillin-clavulanate, cefotaxime, ceftriaxone, and fluoroquinolones) in patients without risk factors for multidrug-resistant (MDR) pathogens in case of early-onset HAP (first 4 days of hospitalization). However, making such choice is not so obvious in ICU patients, and adherence to guidelines is associated with a high rate of unnecessary broad-spectrum antibiotics (Roquilly et al., 2016; Ekren et al., 2018).

Microbiological confirmation of HAP is a crucial step for tailoring antibiotic therapy. Nevertheless, current culture methods take 48–72 h to obtain antimicrobial susceptibility results. Moreover, traditional techniques fail to recover pathogens in up to 30% of clinically-diagnosed HAP (Roquilly et al., 2019). Recently, syndromic multiplex molecular tests have emerged as powerful tools for rapid diagnostics (meningitis/encephalitis, gastroenteritis, bacteraemia, pneumonia) (Couturier and Bard, 2019; Poole and Clark, 2020). Initially based on qualitative DNA detection, those approaches were not suitable for diagnosing pneumonia caused by common colonizers of the upper airways (e.g., *Streptococcus pneumoniae*, *Haemophilus influenzae*). The FilmArray^®^ Pneumonia *plus* Panel (FAPP) is a new panel for HAP, which offers potential advantage to detect and quantify in a single test, 27 respiratory pathogens (18 bacteria, 9 viruses) and 7 antibiotic resistance genes.

The aim of this study was to assess the performances of this new molecular test on bronchoscopy specimens [bronchoalveolar lavages (BAL) and/or endotracheal aspirates (ETA)] from 100 ICU patients with HAP requiring mechanical ventilation.

## Materials and Methods

### Ethic and Study Design

The study protocol was approved by our local Ethical Committee (GNEDS, Nantes, France). Patients and relatives were informed of the trial. Consent was waived according to French law.

### Population and Specimen Collection

The study was conducted at the Nantes University Hospital (France), in 3 ICUs located on two sites spaced 10 km apart. We recruited 100 critically ill adult patients receiving mechanical ventilation with clinically suspected HAP, between October 2018 and January 2020 (Table 1). Pneumonia was suspected based on European guidelines, if there were the following criteria: a new or persistent radiological pulmonary infiltrate without another obvious cause combined with two clinical signs among fever, purulent endotracheal secretions, hyperleukocytosis or leukopenia, and increasing oxygen requirements (Torres et al., 2017). Patients underwent a bronchoscopy with BAL and/or ETA at the time of suspicion of HAP (BAL_DS (for_ _D__IAGNO__S__IS)_ and ETA_DS_, respectively). In addition, if an ETA was collected 2–3 days later, as part of a routine clinical care, the specimen [ETA_TT(for_ _T__REA__T__MENT)_] was also sent to the laboratory for microbiological analysis. A total of 237 respiratory specimens were analyzed (76 BAL_DS_, 82 ETA_DS_, and 79 ETA_TT_). Both BAL_DS_ and ETA_DS_ were collected in 58 patients.

**TABLE 1:** Characteristics of patients at onset of pneumonia.

**Patient characteristics**	***n* = 100**
Age (years), Median (range)	57 (19–85)
Male sex, *n* (%)	81 (81%)
Median length of hospital stay before pneumonia, days	6
Median length of ICU stay before pneumonia, days	5
Early-onset pneumonia, *n* (%)	33 (33%)
Late-onset pneumonia, *n* (%)	67 (67%)
Ventilator-associated pneumonia, *n* (%)	87 (87%)
Most pejorative PaO_2_/FiO_2_ at day 1, Median (range)	135 (56–309)
Risk factors for MRSA^a^, *n* (%)	6 (6%)
Previous isolation of ESBL-Enterobacteria, *n* (%)	2 (2%)
Antibiotics use during the previous 90 days, *n* (%)	40 (40%)
Antibiotics use before sampling at the time of HAP diagnosis	25 (25%)

*^*a*^Recent colonization by MRSA, chronic skin lesions, chronic renal replacement therapy.*

### Microbiological Testing

The respiratory specimens were analyzed in parallel by routine microbiology testing and FAPP, as soon as they arrived at the microbiology laboratory. The turnaround times from samples to validated results were recorded. Results of routine microbiology testing were analyzed independently of FAPP.

#### Routine Microbiology Testing

Gram staining and bacteriological cultures were performed for all respiratory specimens according to the French REMIC recommendations (Société Française de Microbiologie [SFM], 2018). Briefly, 10 μL of the samples were seeded directly (for BAL), or after dilution (1:100 after fluidification for ETA), onto Columbia horse blood (Oxoid), Chocolate agar (BD), and chromogenic UriSelect4 agar (Biorad) plates, and if necessary (cases of chronic obstructive pulmonary disease) on Chapman (bioMérieux) and Cetrimide (Biorad) selective agar plates, then incubated at 37°C in 5% CO_2_ for 24–48 h, as necessary. Plates were examined daily for bacterial growth. *Streptococcus pneumoniae*, *Haemophilus influenzae*, *Staphylococcus aureus*, Enterobacteriales, *Pseudomonas aeruginosa*, *Stenotrophomonas maltophilia*, and any other largely predominant pathogen were searched on the plates. In accordance with current guidelines, the positivity thresholds were 10^5^ CFUs/mL for ETA and 10^4^ CFUs/mL for BAL, but in BAL, potential pathogens that were present in pure culture at 10^3^ CFUs/mL and associated with many leukocytes at Gram staining were reported as positive. Culture results were considered as negative if there was no significant growth or a normal non-pathogenic flora. Bacterial isolates were identified by mass spectrometry (BioMérieux). Antimicrobial susceptibility testing (AST) was performed according to the CA-SFM/EUCAST guidelines (Société Française de Microbiologie [SFM], 2019), using Vitek2 AST cards. Based on phenotypic susceptibility results, additional tests were performed if required, for ESBLs (Mastdiscs™ D68C), carbapenemases (CORIS BioConcept RESIST-3 O.K.N. immuno-chromatographic test and/or in-house real-time PCRs for the *bla*_KPC_, *bla*_OXA–48_, *bla*_VIM_, *bla*_IMP_, and *bla*_NDM_ genes), and methicillin-resistance detection (Alere™ PBP2A culture colony test and BDMAX™ StaphSR). Furthermore, when requested by the Clinicians, the presence in respiratory samples of *Mycoplasma pneumoniae* and/or respiratory viruses was investigated by real-time PCR (Fast Track Diagnostics^®^ Respiratory Pathogens 21 qPCR assay for viruses and/or in-house real-time PCR for *M. pneumoniae*).

#### FilmArray Pneumonia *Plus* Panel Assay

The BioFire^®^ FilmArray^®^ Pneumonia *plus* Panel (bioMérieux) was performed according to the manufacturer’s instructions, with a handling time of ∼5 min. Briefly, the respiratory sample collected with a flocked swab (∼ 200 μL) and then mixed with a sample buffer, was injected along with an hydration solution in the reagent pouch “Pneumonia *plus* Panel,” which was then inserted into the FilmArray^®^ instrument. The test consisted of automated nucleic acid extraction, purification, amplification, detection, and analysis with each target reported as “detected” or “not detected.” A semi-quantitative measurement reported into bins (i.e., 10^4^, 10^5^, 10^6^, and ≥ 10^7^ bacterial DNA copies/mL) was provided for 15 bacteria, if detected. The panel included 15 bacteria, 3 atypical bacteria, 9 viruses, and 7 antimicrobial resistance genes (Table 2). Each resistance marker was reported only if the potential microorganism harboring the gene was concomitantly detected in the sample. Clinicians were left blinded to the FAPP results.

**TABLE 2:** FilmArray^®^ pneumonia *plus* panel targets.

**Variables**
**15 Bacteria reported into bins (10^4^, 10^5^, 10^6^, and ≥ 10^7^ DNA copies/mL)**
*Acinetobacter calcoaceticus baumannii* complex
*Enterobacter cloacae* complex
*Escherichia coli*
*Haemophilus influenza*
*Klebsiella aerogenes*
*Klebsiella oxytoca*
*Klebsiella pneumoniae* group
*Moraxella catarrhalis*
*Proteus* spp.
*Pseudomonas aeruginosa*
*Serratia marcescens*
*Staphylococcus aureus*
*Streptococcus agalactiae*
*Streptococcus pneumoniae*
*Streptococcus pyogenes*
**3 Atypical bacteria**
*Chlamydophilia pneumoniae*
*Legionella pneumophila*
*Mycoplasma pneumoniae*
**9 Viruses**
Adenovirus
Coronavirus (229E, OC43, HKU1, NL63)
human Metapneumovirus
Influenza A
Influenza B
MERS CoV
Parainfluenza viruses
Rhinovirus/Enterovirus
RSV
**7 Antimicrobial resistance genes**
MRSA genes (*mecA/C* and MREJ)
Carbapenemases (*bla*_KPC_, *bla*_NDM_, *bla*_OXA–48–like_, *bla*_VIM_, *bla*_IMP_)
ESBL (*bla*_CTX–M_)

### Data Analysis

BAL were considered as positive with FAPP when at least one microbial target was detected (at ≥10^4^ copies/mL for semi-quantified bacteria). For ETA, in order to match the culture threshold that differentiate commensalism from pathogenicity (≥10^5^ CFUs/mL), we set up a bin threshold of ≥10^5^ copies/mL to consider the 15 semi-quantitative bacterial targets as positive. The agreement between FAPP and culture was measured for each bacterial pathogen in the form of negative percent agreement (NPA), positive percent agreement (PPA) and overall percent agreement (OPA), and their two-sided 95 percent confidence intervals. In order to explain discrepant results, cultures were reread after routine final reports in light of results obtained with FAPP. Concordance was calculated based on the original culture reading.

## Results

### Summary of FAPP Findings

**FIGURE 1:**
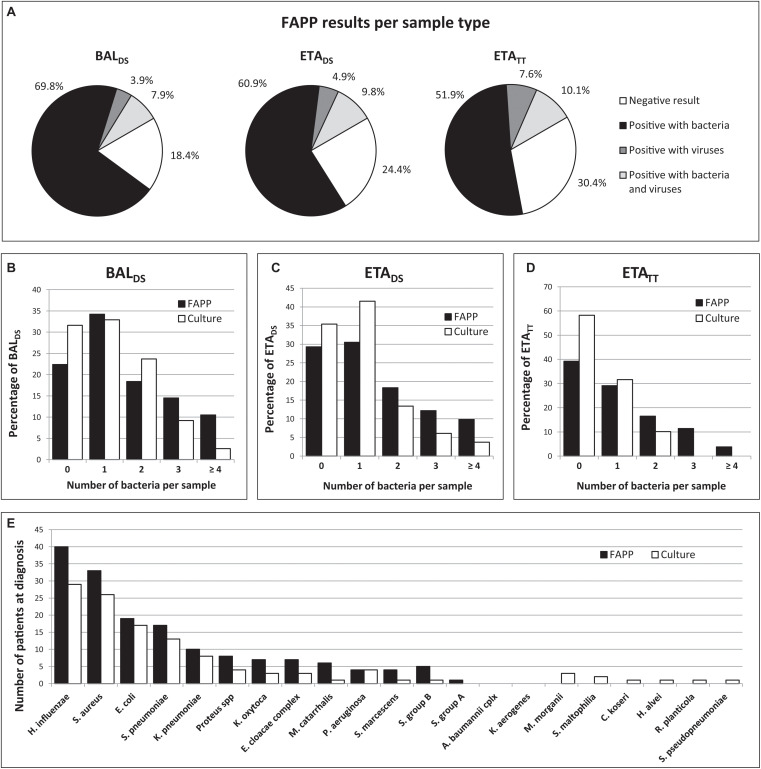
Summary of FAPP and culture results. FAPP results distribution per pathogen category and sample type **(A)**. Number of bacteria per sample with FAPP compared to culture in BAL_DS_ **(B)**, ETA_DS_ **(C)**, and ETA_TT_ **(D)**. FAPP results compared to culture results for bacterial detection **(E)**.

**FIGURE 2:**
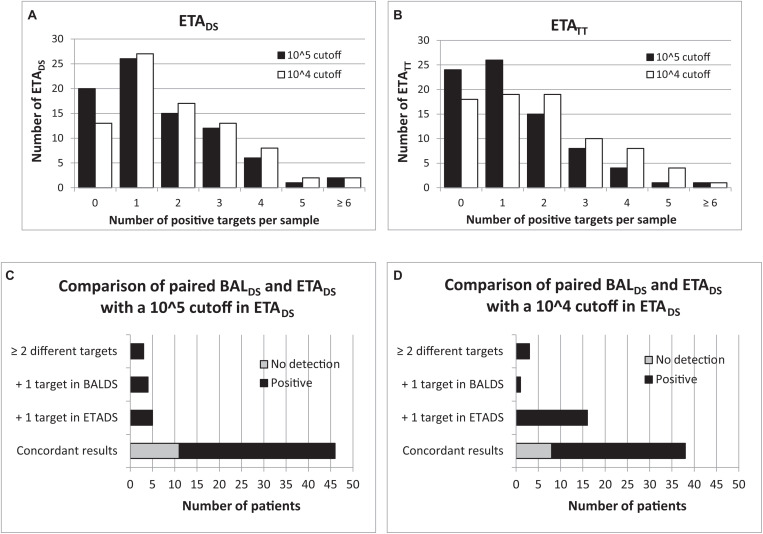
Comparison of 10^4^ and 10^5^ cutoffs with FAPP for ETA. Number of positive detections per sample for each threshold, in ETA collected at diagnosis **(A)** and 2–3 days later **(B)**. Comparison of paired BAL_DS_ and ETA_DS_ for each threshold **(C,D)**.

At the time of HAP diagnosis, FAPP yielded positive results with significant levels (i.e., ≥ 10^4^ bin in BAL and ≥ 10^5^ bin in ETA for semi-quantified bacteria) in 82/100 patients. Thus, as shown in Figure 1A, 81.6% (62/76) BAL_DS_, and 75.6% (62/82) ETA_DS_ were positive for at least one target. Of these, more than half were positive for at least two pathogens (36/62 (58.1%) for BAL_DS_, and 36/62 (58.1%) for ETA_DS_), leading to the diagnosis of coinfection in 49/100 patients (Figure 1). Multiple detections per positive specimen were not higher in ETA_DS_ than in BAL_DS_, since bacteria with bin results of 10^4^ were considered as negative in ETA (it represented 23 bacteria in 21 ETA_DS_). Of note, if the 10^4^ cutoff had been used for ETA, 84.1% (69/82) ETA_DS_ would have been positive, and multiple targets would have been detected in 60.9% (42/69) of these specimens (Figure 2). A maximum of 7 pathogens (6 bacteria and one human rhinovirus/enterovirus) was detected in one patient (BAL_DS_ and ETA_DS_). The most common pathogens detected at diagnosis were *H. influenzae*, *S. aureus*, *E. coli*, *S. pneumoniae*, and *K. pneumoniae*, which were found in 40 (40%), 33 (33%), 19 (19%), 17 (17%), and 10 (10%) patients, respectively (Figure 1). The panel identified 6 viruses at diagnosis [human rhinovirus/enterovirus (5 patients), coronavirus (4 patients), influenza A (3 patients), adenovirus (2 patients), parainfluenza viruses (2 patients), and RSV (1 patient)] in 16/100 patients (11.8% (9/76) BAL_DS_, and 14.6% (12/82) ETA_DS_). In most cases, it corresponded to viral-bacterial co-infections (12 patients, including one with multiple viruses (adenovirus and influenza A) and *S. pneumoniae*) (Supplementary Table S1). An atypical bacteria (*M. pneumoniae*) was detected with other bacteria in one patient. The positivity rate of ETA_TT_ obtained during follow-up was 69.6% (55/79), and 38 bacteria were below the 10^5^ cutoff in 29 ETA_TT_ (Figures 1A, 2). Four types of resistance genes were detected in 8 patients: *mecA/C* and MREJ (one patient), and the CTX-M ESBL (7 patients), either alone (5 patients) or combined with a carbapenemase (*bla*_NDM_ in one patient, and *bla*_OXA–48–like_ in one another). The median turnaround time (from sample collection to results) was 4 h 15 min (BAL_DS_ or ETA_DS_).

### Summary of Routine Microbiology Testing

At HAP diagnosis, culture identified one or more bacteria in 73/100 patients (52/76 (68.4%) BAL_DS_ and 53/82 (64.6%) ETA_DS_), and respiratory viruses were detected in 8/35 (22.9%) patients who benefited from a Fast Track multiplex PCR routinely ordered by clinicians, yielding an overall positive detection in 78/100 patients. Two or more bacterial pathogens were identified and reported in 32/100 patients, in a higher proportion of BAL_DS_ (27/52, 51.9%) than ETA_DS_ (19/53, 35.8%), certainly because BAL are more distal than ETA and are normally not contaminated. Indeed, this property might have encouraged microbiologists to identify and report any bacteria found in these distal specimens rather than concluding to “polymicrobial flora.” Thus, only 25.0% (6/24) of culture-negative BAL_DS_ had results reported as “mixed bacterial flora” vs. 41.4% (12/29) of culture-negative ETA_DS_ (Supplementary Table S1). The most frequent bacteria detected by culture were *H. influenzae*, *S. aureus*, *E. coli*, *S. pneumoniae*, and *K. pneumoniae* in 29 (29%), 26 (26%), 17 (17%), 13 (13%), and 8 (8%) patients, respectively (Figure 1). Culture showed a lower positivity rate of 41.8% (33/79) for ETA_TT_ collected during follow-up, with a high proportion of culture-negative results reported as “no growth” (35/46, 76.1%) (Supplementary Table S1). Regarding AST, *Enterobacteriaceae* resistant to third-generation cephalosporins were found on average 2 days after specimens collection, in 11/100 patients. In 7 cases, it was ESBL-producing strains (*K. pneumoniae* or *E. coli*), while in the 4 others cases, high-level cephalosporinases were confirmed with additional tests (Mastdiscs™ D68C), in strains of *E. cloacae* complex (2 patients), *S. marcescens* (1 patient), and *E. coli* (1 patient). Two ESBL-producing *K. pneumoniae* that were resistant to ertapenem ± imipenem, were also confirmed to be carbapenemase (NDM or OXA-48 like) producers, by means of an immuno-chromatographic test (CORIS BioConcept RESIST-3 O.K.N.) performed 2 days after specimen collection. All strains of *P. aeruginosa* detected in 4 patients were susceptible to ceftazidime. The mean turnaround time from sample collection to results validation was 70 h for BAL_DS_, and 64 h for ETA_DS_.

### Comparison of FAPP and Routine Microbiology Testing

**FIGURE 3:**
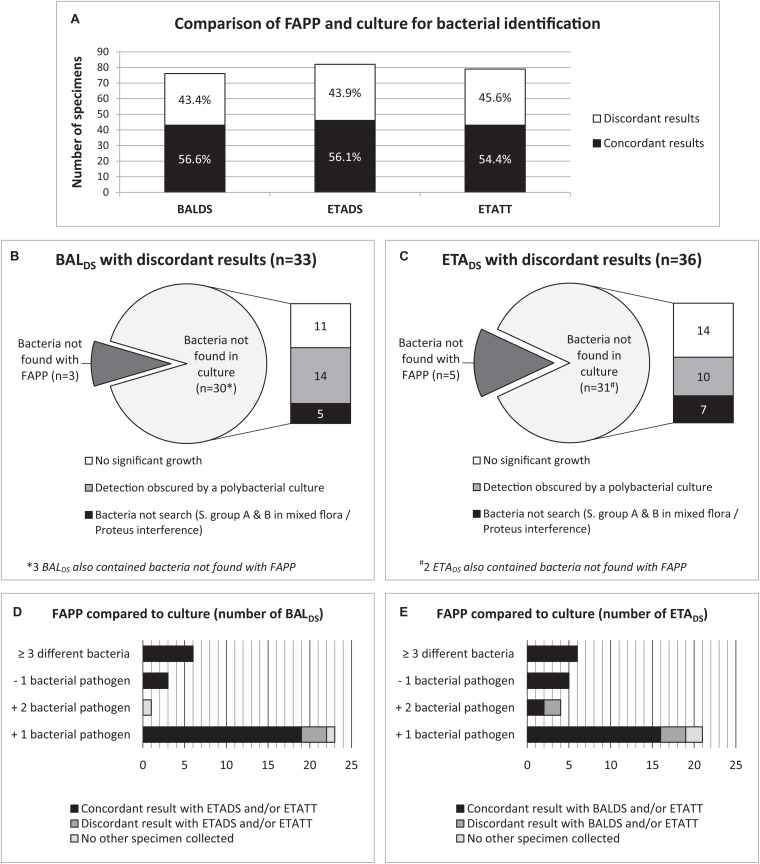
Analysis of discordant results between FAPP and culture at diagnosis, by rereading cultures in light of FAPP results, and by investigating the concordance with FAPP results from the paired ETA_DS_/BAL_DS_ and/or ETA_TT_ if collected. Concordance and discordance between FAPP and culture identifications **(A)**. Causes of discordant results in BAL_DS_ **(B)** and ETA_DS_ **(C)**. Discrepancy investigations in BAL_DS_ **(D)** and ETA_DS_ **(E)**.

In total, at HAP diagnosis, just over half of the specimens were concordant for the bacterial identification (43/76 (56.6%) BAL_DS_ and 46/82 (56.1%) ETA_DS_) (Figure 3 and Table 3). In most of the discordant specimens (23/33 (69.7%) BAL_DS_ and 21/36 (58.3%) ETA_DS_), FAPP identified one supplemental bacterial pathogen, which was most often confirmed by FAPP in the paired respiratory sample and/or in the ETA_TT_ collected 2–3 days later (Figure 3). By rereading the plates in light of FAPP results after final report, we showed that an absence of significant growth, or polybacterial cultures impeding the accurate visualization of non-predominant pathogens, explained almost equally the majority of the non-detections in culture (Figure 3). In the rest of the cases, the corresponding bacteria had not been searched on the plates (*S. pyogenes* or *S. agalactiae* in mixed flora, or because of an impossibility due to *Proteus* invasion) (Figure 3). Furthermore, in 8 patients [5/76 (6.6%) BAL_DS_ and 6/82 (7.3%) ETA_DS_], culture yielded bacteria that were not targeted by FAPP (*Citrobacter koseri*, *Hafnia alvei*, *Morganella morganii*, *Raoultella planticola*, *Stenotrophomonas maltophilia*, and *Streptococcus pseudopneumoniae*), and two FAPP false-negative results were observed: *K. oxytoca* (one BAL_DS_ with a pure culture at 10^3^ CFUs/mL), and *H. influenzae* (one polymicrobial ETA_DS_ with *H. influenzae* at > 10^5^ CFUs/mL) (Figure 1E and Table 3). The atypical bacteria *M. pneumoniae* found in one patient with FAPP, had not been searched with conventional methods at the time of HAP diagnosis, but was subsequently confirmed with an in-house real-time PCR. The performance data for each FAPP bacterial target are provided in Table 3. The overall percent agreement (OPA) between FAPP and culture results ranged from 88 to 100% in BAL_DS_, and 82 to 100% in ETA_DS_. Only three bacterial species of the panel had an OPA bellow 95%: *H. influenzae* and *S. aureus* in BAL_DS_ and ETA_DS_, and *K. oxytoca* in BAL_DS._

**TABLE 3:** Comparison of FAPP and culture for each FAPP bacterial target, at the time of HAP diagnosis.

**FAPP Bacterial targets**	**BAL_DS_ (number of results for FAPP/culture)**							**ETA_DS_(number of results for FAPP/culture)**						
		
	**(+ / +)**	**(+ /−)**	**(−/ +)**	**(−/−)**	**PPA (95% CI)**	**NPA (95% CI)**	**OPA (95% CI)**	**(+ / +)**	**(+ /−)**	**(−/ +)**	**(−/−)**	**PPA (95% CI)**	**NPA (95% CI)**	**OPA (95% CI)**
*A. baumannii*	0	0	0	76	NA	100 [100; 100]	100 [100; 100]	0	0	0	82	NA	100 [100; 100]	100 [100; 100]
*E. cloacae*	2	3	0	71	100 [100; 100]	96 [92; 100]	96 [92; 100]	2	3	0	77	100 [100; 100]	96 [92; 100]	96 [92; 100]
*E. coli*	15	2	0	59	100 [100; 100]	97 [94; 100]	97 [94; 100]	9	0	0	73	100 [100; 100]	100 [100; 100]	100 [100; 100]
*H. influenzae*	23	9	0	44	100 [100; 100]	83 [73; 93]	88 [81; 95]	20	14	1	47	95 [86; 100]	77 [66; 88]	82 [73; 90]
*K. aerogenes*	0	0	0	76	NA	100 [100; 100]	100 [100; 100]	0	0	0	82	NA	100 [100; 100]	100 [100; 100]
*K. oxytoca*	2	4	1	69	67 [13; 100]	95 [89;100]	93 [88; 99]	2	4	0	76	100 [100; 100]	95 [90; 100]	95 [90; 100]
*K. pneumoniae*	7	2	0	67	100 [100; 100]	97 [94; 100]	97 [94; 100]	6	1	0	75	100 [100; 100]	99 [96; 100]	99 [96; 100]
*M. catarrhalis*	0	2	0	74	NA	97 [94; 100]	97 [94; 100]	1	4	0	77	100 [100; 100]	95 [90; 100]	95 [90; 100]
*Proteus* spp.	1	4	0	71	100 [100; 100]	95 [90; 100]	95 [90; 100]	4	4	0	74	100 [100; 100]	95 [90; 100]	95 [90; 100]
*P. aeruginosa*	3	0	0	73	100 [100; 100]	100 [100; 100]	100 [100; 100]	3	0	0	79	100 [100; 100]	100 [100; 100]	100 [100; 100]
*S. marcescens*	1	1	0	74	100 [100; 100]	99 [96; 100]	99 [96; 100]	0	3	0	79	NA	96 [92; 100]	96 [92; 100]
*S. aureus*	17	6	0	53	100 [100; 100]	90 [82; 98]	92 [86; 98]	19	5	0	58	100 [100; 100]	92 [85; 99]	94 [89; 99]
*S. agalactiae*	1	3	0	72	100 [100; 100]	96 [92; 100]	96 [92; 100]	0	4	0	78	NA	95 [90; 100]	95 [90; 100]
*S. pneumoniae*	11	4	0	61	100 [100; 100]	94 [88; 100]	95 [90; 100]	9	4	0	69	100 [100; 100]	95 [90; 100]	95 [90; 100]
*S. pyogenes*	0	1	0	75	NA	99 [96; 100]	99 [96; 100]	0	0	0	82	NA	100 [100; 100]	100 [100; 100]
Total	83	41	1	1015	99 [96; 100]	96 [95; 97]	96 [95; 97]	75	46	1	1108	99 [96; 100]	96 [95; 97]	96 [95; 97]

**FIGURE 4:**
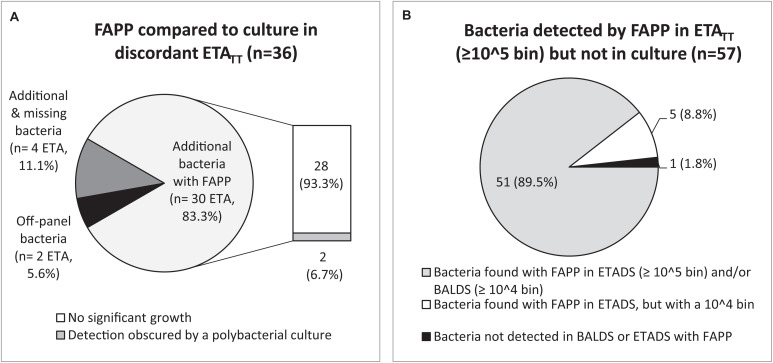
Analysis of discordant results between FAPP and culture in ETA_TT_. Causes of discordant results in ETA_TT_ **(A)**. Discrepancy investigations for each additional bacteria detected by FAPP **(B)**.

Regarding the 79 ETA_TT_ obtained under treatment, 45.6% (36/79) had discordant results between both methods (Figures 3A, 4 and Supplementary Table S1). Not surprisingly, most discrepancies (28/36, 77.8%) were explained by no growth of bacteria identified with FAPP (Figure 4A). The vast majority of the 57 FAPP-positive bacterial targets that were not reported by routine culture, had already been detected by FAPP at diagnosis, either above positive threshold values (51/57, 89.5%), or not (bin result of 10^4^ in ETA_DS_) in a few cases (5/57, 8.8%) (Figure 4B).

Regarding FAPP semi-quantitative results, most bacteria with bin results of 10^4^ in ETA (i.e., below our positivity threshold) were not reported in culture (23/23 (100%) in ETA_DS_, and 36/38 (94.7%) in ETA_TT_). On the other hand, for patients with ETA at diagnosis and 2–3 days later, 38.9% (7/18) of the detections with a bin value of 10^4^ in ETA_DS_ were positive again in ETA_TT_ with a higher bin value (≥ 10^5^ copies/mL). No linear relationship was observed between the bin and CFUs/mL variables (Supplementary Table S1). However, semi-quantitative culture results were not stratified into log_10_ ranges above positive thresholds (10^5^ CFUs/mL for ETA and 10^4^ CFUs/mL for BAL).

Eighteen resistance markers were detected with FAPP in 15 samples (2 *mecA/C* and MREJ, 13 *bla*_CTX–M_, 2 *bla*_NDM_, and 1 *bla*_OXA–48–like_) (Supplementary Table S1). All ESBL and carbapenemases were confirmed by standard laboratory protocols (AST and additional tests performed in routine). Among both methicillin-resistant *S. aureus* (MRSA) detected with FAPP, one found at 10^4^ bin in ETA_TT_ did not grow in culture. The other corresponded to a false-positive *mecA/C* and MREJ result since a methicillin-susceptible *S. aureus* (MSSA) was found in culture. This result was repeatable after retesting with FAPP, but none of the comparator methods (BDMAX™ StaphSR performed on the same BAL_DS_, or Alere™ PBP2A testing and cefoxitin susceptibility testing performed on several colonies) found a MRSA. No additional cases of methicillin-resistance, ESBL, or carbapenemase production were found with routine microbiology testing.

Lastly, based on FAPP results, an initial antibiotic therapy by amoxicillin-clavulanate could have been proposed in 83/100 patients, whose results ruled out pathogens with chromosomally-encoded cephalosporinase (i.e., *P. aeruginosa*, *A. baumannii*, *E. cloacae* complex, *K. aerogenes*, and *S. marcescens*) and/or resistance markers of the panel. However, this antibiotic would have not been optimal in 7/83 (8.4%) patients. In fact, in those cases, culture brought to light bacterial strains with acquired resistance to amoxicillin-clavulanate (2 *H. influenzae* and 2 *E. coli*, in 4 patients), or pathogens not targeted by FAPP and naturally resistant to amoxicillin-clavulanate (1 *H. alvei*, 1 *M. morganii*, and 1 *S. maltophilia*, in 3 patients). A medico-economic evaluation is ongoing to determine what impacts FAPP results would have had on care and antibiotics prescribing (Guillotin et al., in preparation).

### Comparison of Paired BALDS and ETADS Specimens

Among the 58 patients with paired BAL_DS_ and ETA_DS_, 46 (79.3%) had the same pathogen(s) (or no pathogen) identified in both samples with FAPP. Of the 12 discrepancies observed, 5 were due to detection of one more pathogen in ETA_DS_ (2 viruses, and 3 bacteria at 10^5^ bin), 4 to detection of one additional bacteria in BAL_DS_ (3 of which were also detected in ETA_DS_, but considered as negative since at 10^4^ bin in ETA_DS_). In the 3 latter cases, the difference relied on two pathogens. If bacteria with a 10^4^ bin had been considered as positive in ETA_DS_, the agreement between both types of specimens would have been less satisfactory, with 38/58 (65.5%) concordant results (Figures 2C,D). Regarding culture, concordant results were obtained in 48/58 (82.8%) paired specimens. In most of the discordant cases (7/10), there was at least one additional pathogen detected in BAL_DS_. At last, only two of all discordant pairs (*n* = 20 with FAPP and/or culture) were confirmed with both methods (similar results between FAPP and culture) (Supplementary Table S1).

## Discussion

At first developed for the detection of widely circulating respiratory viruses and selected atypical bacteria, syndromic molecular tests for respiratory tract infections continuously expand their breadth of coverage to improve diagnostic accuracy. FAPP and the Curetis^®^ Unyvero Hospitalized Pneumonia Panel, are the first two, FDA approved and CE marked, commercially available platforms which target a large number of lower respiratory tract pathogens and resistance genes from aspirates or BAL fluids (Collins et al., 2020; Murphy et al., 2020). There are no published prospective studies comparing the performances of both plateforms, but regarding their technical characteristics, FAPP offers a shorter turnaround time (75 min vs. 4–5 h), a smaller footprint, and the possibility to detect viral pathogens and to semi-quantify bacteria (Poole and Clark, 2020). In this study, this test was compared to routine microbiological methods using 237 prospectively collected BAL and ETA specimens obtained from 100 ICU patients at the time of suspected HAP and, if possible, at a later timepoint during follow-up.

As expected, implementation of FAPP shortened the delay in getting results (4 h 15 min on average, one ICU setting being located 10 km away from the laboratory vs. 64–70 h with culture). In accordance with recent evaluations (Lee et al., 2019; Buchan et al., 2020; Murphy et al., 2020; Yoo et al., 2020), FAPP increased the positivity rate of diagnostic testing (81.6% for BAL_DS_, and 75.6% for ETA_DS_), enabling identification of additional bacteria in 39.5% BAL_DS_ and 37.8% ETA_DS_. The most common pathogens detected were consistently the same across both methods (i.e., in order of prevalence, *H. influenzae*, *S. aureus*, *E. coli*, *S. pneumoniae*, and *K. pneumoniae*). This pathogen distribution, which mostly corresponded to bacterial species that are part of the normal throat flora, was not really different from that described in community-acquired pneumonia. According to the latest European surveillance report on healthcare-associated infections acquired in ICU in 2017, *P. aeruginosa* was the most common microorganism associated with pneumonia (19.9%), followed by *S. aureus* (18.5%), *Klebsiella* spp. (15.2%), and *E. coli* (13.5%). In the majority of cases, pneumonia was associated with intubation, and HAP episodes occurred after an average length of ICU stay of 7.3–12.1 days, depending on the country (European Centre for Disease Prevention and Control [ECDC], 2019). In our study, whatever the method used, *P. aeruginosa* was identified in only 4/100 patients, including three who did not present classic risk factors for MDR pathogens (no previous antimicrobial therapy or hospitalization in the preceding 90 days, and length of ICU stay of 4–6 days) (Torres et al., 2017; European Centre for Disease Prevention and Control [ECDC], 2019). The most common pathogen of our study, *H. influenzae*, was detected with FAPP in 40/100 patients at diagnosis, after a median length of ICU stay of 4 days, but was less frequently found in culture (29/100 patients). In line with our data, the majority of discrepancies previously reported between FAPP and culture, concerned the same fastidious bacteria, and were explained by the higher sensitivity of the molecular test and/or antibiotics consumption before sampling (Lee et al., 2019; Yoo et al., 2020). Here, in just over half of the discrepant cases, *H. influenzae* grew on the enriched medium used for culture, but was overgrown by other pathogens or commensal bacteria, and was therefore not detected and/or not reported. Thus, whether detection of *H. influenzae* represents true infection or colonization will be an important area for future research. It is less a question for *S. aureus*, which is a member of the normal nasal flora in about 30% of the population, but can also be regarded as an aggressive and life-threatening bacterial pathogen (Laux et al., 2019). However, in the same manner as for *H. influenzae*, discrepant results obtained for *S. aureus* in 7 patients (FAPP-positive but culture-negative), were not always explained by no bacterial growth. As noted previously, these findings pointed the limits of bacterial cultures, which are subject to interpretation and based on selection of dominant species assigned to play a pathogenic role, the minority species being not considered (Buchan et al., 2020; Murphy et al., 2020). These results confirmed the need to inoculate selective agars for enhancing detection of specific bacteria in lower airways (Chapin and Doern, 1983; Doern and Brogden-Torres, 1992). Moreover, a significant part of discrepancies was linked to a lack of growth in culture [11/33 (33.3%) for BAL_DS_, 14/36 (38.9%) for ETA_DS_, and 28/36 (77.8%) for ETA_TT_]. A quater (25/100) of the patients enrolled in the study had received antibiotics before sampling at the time of HAP diagnosis, while ETA_TT_ were collected under antibiotic treatment. Thus, in our view, these culture-negative detections most likely corresponded to pathogens present at low abundances (i.e., below the limit of detection in culture) or to remnant DNA from non-viable bacteria, notably in supplemental ETA_TT_, rather than non-specific amplifications. In fact, FAPP results from ETA_TT_ and/or paired BAL_DS_ or ETA_DS_ allowed to verify a lot of FAPP-positive results for bacteria that had been undetected by culture. As a result, FAPP may prove useful to guide treatment in situations of diagnostic uncertainty where patients have received antibiotics before sampling, and/or have unfavorable treatment outcomes after obtaining culture, because the higher sensitivity of this method decreases the likelihood to miss out on pathogens of the panel.

An important finding of this study, was that the implementation of FAPP increased the number of coinfections detected compared to conventional methods. Thus, the multiplex panel identified mixed infections in 49/100 patients (58.1% of positive BAL_DS_ or ETA_DS_), compared to 32/100 patients (51.9% and 34.6% of positive BAL_DS_ and ETA_DS_, respectively) by culture. These data corroborate other published results, and outline that the true incidence of polymicrobial HAP is probably underestimated with conventional techniques (Lee et al., 2019; Buchan et al., 2020; Murphy et al., 2020; Yoo et al., 2020). It remains to be evaluated whether detection of more pathogens will increase cure rates, and not adversely result in unnecessary consumption of broad-spectrum antimicrobials. New research avenues have emerged in recent years about the pathophysiology of HAP, because their rate of clinical cure does not commonly exceed 70% (Roquilly et al., 2019). It has been demonstrated that healthy lungs harbor a diverse and dynamic microbiota, which is profoundly altered in critically ill patients, and would play a role in the development of pneumonia. Future progress in this field should help understand how to appreciate lower abundance taxa of the microbiome, over other most numerous species (Panzer et al., 2018; Roquilly et al., 2019).

In our study, viruses were identified in 16/100 patients with FAPP, but in half of them no viral testing had been ordered, including one with an influenza A. As this virus can be responsible for severe pneumonia, and can represent a potential source of intra-hospital transmission, FAPP demonstrated a concrete benefit in that case (Loubet et al., 2017; Van Someren Gréve et al., 2018). Conventional testing for respiratory viruses other than influenza, has not been universally embraced as a standard of care, especially because viral carriage is not uncommon in patients with HAP (Loubet et al., 2017; Torres et al., 2017; Papazian et al., 2020). Furthermore, while the interaction between influenza and *S. pneumoniae* or *S. aureus* is a major contributor to influenza mortality in community-acquired pneumonia, the consequences of viral-bacterial coinfection on the prognosis of HAP is still unclear (Loubet et al., 2017; Van Someren Gréve et al., 2018). In our study, the majority (75%) of the 16 patients with identified viruses, were coinfected with bacteria, and 4 patients were infected with a single virus (influenza A, RSV, coronavirus, or human rhinovirus/enterovirus). Furthermore, in our opinion, additional viral targets (herpes simplex virus and cytomegalovirus) might be relevant if added to the panel, because reactivation of these viruses are indeed quite frequent in ICU patients, causing nosocomial viral pneumonia that can evolve into acute respiratory distress syndrome (ARDS) (Papazian et al., 2020).

One special feature of FAPP, is its ability to provide semiquantitative assessment of bacterial DNA targets to help in interpretation. Here, we showed that in BAL, 10^4^ copies/mL corresponded to bacterial counts of ∼10^3^–10^4^ CFU/mL. In ETA, bacteria with bin results of 10^4^ copies/mL were not found in culture in 96.7% of the cases (59/61). However, a small proportion (38.9%) of targets quantified as 10^4^ copies/mL in ETA_DS_, were recovered later with higher bin values in ETA_TT_. Thus, we show that in those potentially contaminated samples, targets quantified as 10^4^ copies/mL by FAPP, can be reported as negative to provide results concordant with those routinely reported by culture, in accordance with current guidelines (≥ 10^5^ CFU/mL) (Buchan et al., 2020). Nonetheless, this raises the important question of whether low concentration culture-negative detections with FAPP are adding value in the care of ICU patients. This issue is discussed in the medico-economic evaluation coupled with this study (Guillotin et al., in preparation). We found no correlation between bin ≥ 10^5^ and culture concentrations in both types of specimens. However, the plating method used in the present study did not allow accurate determination of relative quantities beyond 10^4^ CFU/mL for BAL, and 10^5^ CFU/mL for ETA.

An originality of this work lies on the inclusion of 58 patients from whom both BAL_DS_ and ETA_DS_ were collected, and could be compared. Latest European and American guidelines for the management of HAP provide divergent recommendations on sampling techniques to prioritize for diagnosis of HAP. While scientific societies from North America place a high value on non-invasive sampling with semiquantitative cultures (i.e., ETA), European guidelines suggest obtaining distal quantitative samples with invasive techniques to improve the accuracy of results, and reduce overutilization of antibiotics (Kalil et al., 2016; Torres et al., 2017). In fact, endotracheal aspirates may overestimate the presence of bacteria, but they can be performed more quickly and simply, with fewer complications and resources. In our study, provided that a 10^5^ copies/mL threshold was applied for ETA, those specimens appeared equally accurate as BAL for the diagnosis of HAP (concordance obtained in 79.3% of patients with FAPP vs. 82.8% patients for conventional culture).

Finally, this study examined if when compared to culture, informations supplied by FAPP would have had positive impacts on antibiotics prescribing. Regarding the adequacy of bacteria targeted by FAPP, five Gram-negative species including three resistant to amoxicillin-clavulanate (*H. alvei*, *M. morganii*, and *S. maltophilia*) in 3/100 patients, were missed by the panel. On the other hand, the molecular test led to an increased identification of respiratory pathogens, and to the rapid detection of some genotypic markers of resistance in 8 patients. Thus, in total, for covering FAPP findings, the narrow-spectrum antibiotic amoxicillin-clavulanate could have been a therapeutic option in the majority of patients (83%). Nonetheless, natural or acquired resistances to amoxicillin-clavulanate would have gone unnoticed in 8.4% of them. All carbapenemase and/or ESBL-producing strains were correctly detected with the multiplex panel (AST agreed with FAPP). However, it should be noted that the overall prevalence of antimicrobial resistance was low in our study, and it should also be kept in mind that a lack of detection of resistance genes does not necessarily means susceptibility to antibiotics as there are resistance mechanisms that are not detected by FAPP (i.e., derepressed or plasmidic cephalosporinases, or non-CTX-M ESBL). Regarding methicillin resistance, consistent with previous observations, we noticed the false-positive detection of *mecA/C* and MREJ genes in one specimen containing a MSSA in culture (Yoo et al., 2020). Since this respiratory sample was polymicrobial, we hypothesized that it could have contained both a methicillin-resistant coagulase-negative *Staphylococcus* carrying *mecA/C*, and a MSSA with an empty SCCmec cassette (thus positive for MRJE) (Murphy et al., 2020).

## Conclusion

In conclusion, our study demonstrates that FAPP provides results at a speed and sensitivity never possible before, and may allow clinicians to make more informed decisions about antibiotics use and isolation of patients. There is still room for improvements in terms of breadth (amoxicillin-clavulanate naturally resistant Gram-negative bacilli), resistance (MRSA), and cost, but this culture-independent technique may achieve more reliable identification of causative agents than culture. There will be a learning curve for physicians to establish how best to use FAPP results in the management of ICU patients with HAP. To achieve maximum benefit from this new molecular test, nuances in results interpretation might be applied on the basis of clinical presentation, timing of initial antimicrobial therapy (fresh vs. post-treatment samples), sampling type (BAL vs. ETA), and local bacterial ecology and resistance patterns. We are currently assessing the impact of this platform on antibiotic use and patients outcome in our hospital, and are evaluating if an algorithm-based treatment plan guided by FAPP would be of great benefit.

## Data Availability Statement

All datasets generated for this study are included in the article/Supplementary Material.

## Ethics Statement

The studies involving human participants were reviewed and approved by the Groupe Nantais d’Ethique dans le Domaine de la Santé (GNEDS). Written informed consent for participation was not required for this study in accordance with the national legislation and the institutional requirements.

## Author Contributions

LC, AR, and SG were involved in all the aspects of the study and were the guarantors for the data. BG, MB, KL, BR, and AR performed the clinical procedures. LC, TD, EP, and SG performed the laboratory procedures. LC, TD, FG, CP, M-AV, and SG analyzed the data. LC and AR wrote the manuscript. All authors contributed to the article and approved the submitted version.

## Conflict of Interest

The authors declare that the research was conducted in the absence of any commercial or financial relationships that could be construed as a potential conflict of interest.

## Supplementary Material

TABLE S1 FAPP and original culture reading results.
